# Environment shapes the fecal microbiome of invasive carp species

**DOI:** 10.1186/s40168-016-0190-1

**Published:** 2016-08-12

**Authors:** Jessica J. Eichmiller, Matthew J. Hamilton, Christopher Staley, Michael J. Sadowsky, Peter W. Sorensen

**Affiliations:** 1 Department of Fisheries, Wildlife, and Conservation Biology, Minnesota Aquatic Invasive Species Research Center, University of Minnesota, Twin Cities, Saint Paul, MN 55108 USA; 2 Department of Soil, Water and Climate, Biotechnology Institute, University of Minnesota, Saint Paul, MN 55108 USA

**Keywords:** Fecal microbiome, Invasive carp, Microbiota, Community structure, 16SrRNA gene

## Abstract

**Background:**

Although the common, silver, and bighead carps are native and sparsely distributed in Eurasia, these fish have become abundant and invasive in North America. An understanding of the biology of these species may provide insights into sustainable control methods. The animal-associated microbiome plays an important role in host health. Characterization of the carp microbiome and the factors that affect its composition is an important step toward understanding the biology and interrelationships between these species and their environments.

**Results:**

We compared the fecal microbiomes of common, silver, and bighead carps from wild and laboratory environments using Illumina sequencing of bacterial 16S ribosomal RNA (rRNA). The fecal bacterial communities of fish were diverse, with Shannon indices ranging from 2.3 to 4.5. The phyla *Proteobacteria*, *Firmicutes*, and *Fusobacteria* dominated carp guts, comprising 76.7 % of total reads. Environment played a large role in shaping fecal microbial community composition, and microbiomes among captive fishes were more similar than among wild fishes. Although differences among wild fishes could be attributed to feeding preferences, diet did not strongly affect microbial community structure in laboratory-housed fishes. Comparison of wild- and lab-invasive carps revealed five shared OTUs that comprised approximately 40 % of the core fecal microbiome.

**Conclusions:**

The environment is a dominant factor shaping the fecal bacterial communities of invasive carps. Captivity alters the microbiome community structure relative to wild fish, while species differences are pronounced within habitats. Despite the absence of a true stomach, invasive carp species exhibited a core microbiota that warrants future study.

**Electronic supplementary material:**

The online version of this article (doi:10.1186/s40168-016-0190-1) contains supplementary material, which is available to authorized users.

## Background

Aquatic invasive species are among the greatest threats to aquatic ecosystems. In particular, species of carp, such as the common, bighead, and silver carp, can consume large quantities of food and disrupt food chains, while potentially out-competing native species and reaching great densities in invaded ranges [1–4]. The common carp is the most widespread invasive fish in the world. It was introduced to the USA over a century ago, and it has gradually spread throughout lakes and rivers. Once established, common carp act as ecosystem engineers, uprooting aquatic vegetation and increasing phosphorus availability, resulting in eutrophication and ecosystem degradation [5, 6]. In contrast, bighead and silver carps, members of a group known as the “Asian carps,” were recently introduced into the USA in the 1970s. Their range, however, is rapidly expanding and now stretches from the lower Mississippi River to its northern reaches and tributaries.

Although the potential ecosystem impacts of silver and bighead carp are not well known, as large filter feeders they could potentially affect native fish species directly by inducing changes in zooplankton communities [7, 8]. While the management of common carp has occasionally been successful through poisoning entire lakes or manipulating weaknesses in their life history characteristics (e.g., manipulation of predators in spawning habitats) [9], no control strategies have been successful in reducing the population of silver and bighead carps. Novel options for control must therefore come through a more thorough understanding of the biology of these invasive fishes.

The microbiome, the collection of microorganisms associated with an animal, is essential for optimal growth and survival of the host species [10–12]. In particular, the digestive tract microbiota plays an integral role in the breakdown of food, provision of energy, vitamin production, and shaping innate immunity. In humans and other mammals, the gut microbiota has been shown to have broad effects on health and behavior [13]. In fish, the gut microbiota has largely been studied in the context of aquaculture in order to identify or examine the effect of probiotics to enhance growth or health [14–16]. A better understanding of the gut microbiome of fishes, however, might reveal potential for control of these invasive species, since dysbiosis of the gut microbiome has been found to contribute to disease manifestation in humans and other vertebrates [17]. Manipulation of gut microbiota to influence health has recently received greater attention in humans through procedures such as fecal microbiota transplantation [18, 19]. Thus, characterizing the gut microbiome of invasive fishes is an important step toward understanding the community that comprises this “hidden organ,” which might be eventually exploited for species control purposes.

Fish possess a gut microbiome that is distinct from other animals and the microbial communities of water and soil [20]. Previous studies have shown that the fish gut microbiome is dominated by members of the phyla *Proteobacteria*, *Firmicutes*, *Bacteroidetes*, *Actinobacteria*, and *Fusobacteria* [20], and both trophic level and salinity predominantly influence the fish gut microbial community [20–22]. While diet can also affect the gut microbiome, the significance and magnitude of the effect are variable [23–25]. The microbiota of prey items has been shown to influence the gut microbiome in three-spined stickleback; however, host genotype exhibited a larger effect [26]. Gut microbiome diversity was inversely related with dietary diversity in two species of freshwater fishes [27], whereas the effect of diet on Trinidadian guppies was negligible [28]. The gut microbiome can also reflect relative preference for cyanobacteria as a food source [29]. In silver carp, the gut microbiome has also been shown to be geographically and temporally variable [29].

Like other vertebrates, fish likely harbor a core microbiome. Roeselers et al. [30] identified a core microbiome of zebrafish through comparison of lab-raised and wild stocks. Further support of this concept was demonstrated in a reciprocal transplant of microbiota between zebrafish and mice [31]. After transplantation, the microbial community gradually shifted to resemble the typical structure of its new host. However, habitat changes, such as the transition from wild to captive environments can lead to dramatic changes in the gut microbiome of fishes, including decreased gut microbiome diversity [25, 28, 32].

Although our understanding of the structure of the fish microbiome has increased in recent years, there are still important gaps in our current knowledge regarding the factors that shape the fish gut microbiome. The advent of metagenomics and high-throughput amplicon sequencing technologies has demonstrated that culture-based studies of the fish microbiome are inherently biased and do not reflect total community diversity [14, 16]. In the first study of carp using high-throughput sequencing, van Kessel et al. [33] found that nearly half of the sequences in captive carp belonged to the phylum *Fusobacteria*, which were conspicuously absent in culture-dependent studies. However, of the studies that have utilized metagenomic approaches to examine the gut microbiome of carp species, only two have included wild fish [22, 29]. In addition, only one of these studies was done in an invaded range. Ye et al. [29] compared the microbiome of silver carp to gizzard shad, a native fish species that is planktivorous, but does not share the same taxonomic order. This study found differences in microbiota between fish species that could be explained by gut morphology and feeding preferences. Additional research is needed to compare the gut microbiome among closely related species of carps in their invaded range and to characterize the core microbiome.

In this study, we characterized the fecal microbiomes of three species of invasive carps (silver, bighead, and common carps) and determined the relationships between microbial community structure and environment, diet, and fish species. The aims of this study were to determine (1) the effect of environment on the microbiome of invasive carps, (2) how microbial communities of fish species differ within environments and the extent to which diet plays a role, (3) to what extent a core microbiome exists among invasive carps, and (4) how differences in microbiome structure among environments and fish species affect inferred bacterial community function.

Our results shed new light on the understanding of the microbiome of invasive carps and highlight the dominant role of the environment in shaping the fish microbiome.

## Methods

### Sample collection

Wild fishes were collected from both river and lake habitats (Table 1). Bighead carp (*Hypophthalmichthys nobilis*), silver carp (*Hypophthalmichthys molitrix*), common carp (*Cyprinus carpio*), and freshwater drum (*Aplodinotus grunniens*) were caught from the Marseilles reach of the Illinois River, IL, USA (41° 21′ 2′′ N, 88° 26′ 15′′ W) in June and August 2013. Freshwater drums were collected for comparison to the carps because they are an abundant co-occurring carnivorous fish, thus, enabling comparisons across trophic levels.

**Table 1:** Description of fishes used in this study

Species	Common name	Habitat	Diet	Number
*Hypophthalmichthys nobilis*	Bighead carp	Laboratory	Algal feed mixture, see [72]	5
*Hypophthalmichthys molitrix*	Silver carp	Laboratory	Algal feed mixture, see [72]	5
*Cyprinus carpio*	Common carp	Laboratory	2.5-mm pellet feed (Oncor Fry, Skretting USA, Tooele, UT)	5
*Cyprinus carpio*	Common carp	Laboratory	Frozen brine shrimp (San Francisco Bay Brand, Newark, CA)	5
*Carassius auratus*	Goldfish	Laboratory	Flake food (Color Tropical Marine, Pentair Aquatic Ecosystems, Apopka, FL)	5
*Hypophthalmichthys nobilis*	Bighead carp	River	NA	19
*Hypophthalmichthys molitrix*	Silver carp	River	NA	20
*Cyprinus carpio*	Common carp	River	NA	16
*Cyprinus carpio*	Common carp	Lake	NA	13
*Aplodinotus grunniens*	Freshwater drum	River	NA	9

*NA* not applicable

Asian carps were field-identified as either bighead or silver carp, and fin clips were taken for SNP analysis to determine species and exclude F_1_ hybrids [34]. An additional 13 common carp were caught from Lotus (44° 52′ 28′′ N, 93° 31′ 48′′ W), Riley (44° 50′ 10′′ N, 93° 31′ 17′′ W), and Susan (44° 51′ 5′′ N, 93° 32′ 27′′ W) lakes in MN, USA, in August and September 2012. Four fish each were collected from Lotus and Riley lakes, while five were collected from Lake Susan.

Laboratory-housed bighead carp (*H. nobilis*), silver carp (*H. molitrix*), common carp (*C. carpio*), and goldfish (*Carassius auratus*) were also used in this study (Table 1). Common carp and goldfish were obtained from a commercial fish hatchery (Osage Catfisheries Inc., Osage Beach, MO; Hunting Creek Fisheries, PA, respectively). Bighead and silver carps were obtained from an experimental research facility (US Geological Survey, Columbia, MO, USA). Goldfish were included for comparison because they are closely related and hypothesized to have originated from a wild population of Prussian carp (*Carrassius gibelio*) and a model organism. All fish were juvenile development stage and had been housed in the laboratory for at least 6 months.

All laboratory fishes were kept at 18–20 °C, within flow-through tanks with constant aeration. Fish were fed ad libitum once daily, and food source was consistent for at least 3 weeks prior to sampling (Table 1). Fish were sampled from at a minimum of three different tanks. All fish were held in accordance with the University of Minnesota’s Institutional Animal Care and Use Committee (IACUC) (Protocol: 1407-31659A).

Fecal specimens from wild fish were collected within 1 h of fish capture, whereas laboratory fishes were anesthetized in an aerated anesthetic bath (0.01 % MS-222; Syndel, CO, USA) prior to handling. Fecal specimens were collected by manual stripping of live or recently deceased fish. Fish were stroked firmly from pelvic fins to anus, and fecal material was collected in a sterile microcentrifuge tube and stored frozen at −20 °C.

### DNA extraction, PCR, and sequencing

DNA was extracted from frozen fecal samples using the QIAamp DNA Stool Mini Kit (Qiagen, Hilden, Germany) according to the manufacturer’s protocol. The V6 hypervariable region of the 16S rRNA gene was amplified as previously described using a mixture of five forward primers and a barcoded reverse primer to amplify triplicate 50-μL reactions containing 25 ng of fecal DNA each [35]. PCR products were visualized on a 2 % agarose gel and purified using the QIAquick Gel Extraction Kit (Qiagen, Hilden, Germany). Replicate purified reactions were pooled, and DNA was quantified using the QuantiFluor-ST and the dsDNA System (Promega, Madison, WI, USA). Purified amplicons were pooled in equal concentrations.

DNA was sequenced using an Illumina HiSeq 2000 platform at the University of Minnesota Genomics Center (Saint Paul, MN, USA) with up to 20 pooled samples per lane for a total of six runs. Paired-end sequences (100-bp read length) were joined as previously described [19]. Sequencing results were submitted to NCBI under project number SRP071816.

### Sequence processing

Sequence reads were processed using the mothur software package version 1.36.1, as previously described [35, 36]. Sequence reads that had ambiguous bases, more than one mismatch to primer sequences, homopolymers > 8 nt, and quality scores < 35 in a 50-nt window were removed. UCHIME [37] was used to identify possible chimeric sequences in mothur using the default parameters and the SILVA database release 102 of bacterial reference sequences [38]. Sequences identified as possible chimeras were subsequently removed from the dataset. Sequences were aligned using the SILVA database [38]. The threshold for aligning the reverse compliment sequence was set to 0.75, and all other settings were set to default parameters. OTUs were clustered by furthest neighbor at a 97 % similarity cutoff. OTUs were classified using a naïve Bayesian classifier and the Ribosomal Database Project (RDP) taxonomic database release 9 and mother training set 9, with a probability cutoff of 60 % [39]. Chloroplast sequences and sequences that were unclassified at the kingdom level were removed, which comprised 0.001 % of total reads. Sequences that had a frequency ≤ 10 were removed from the dataset [40], removing a total of 1.7 % of reads. The number of reads from each sample was normalized to 150,000 from a maximum of 1,841,796 by randomly subsampling.

### Sequence analysis

The Shannon index of diversity (alpha diversity) was calculated for each experimental group (Table 1) using mothur. One-way ANOVA and Tukey post hoc tests were used to examine differences in OTUs observed (S_obs_) and Shannon index of diversity using JMP, Version 10 (SAS Institute Inc., Cary, NC). The distances among environment-species groups were calculated based on Bray-Curtis dissimilarity [41]. The resulting distances were used to perform a hierarchical clustering of experimental groups using Unweighted Pair Group Method (UPGMA). Results were graphed using TreeView v 1.6.6. Bray-Curtis dissimilarity distances were calculated among individual samples and used to perform ordination and statistical tests. Ordination was done using non-metric multidimensional scaling (NMDS) in two dimensions [42]. NMDS was done separately for lab and wild fishes. Ten iterations were performed, and the iteration resulting in the lowest stress was plotted. Analysis of molecular variance (AMOVA) was used to test for significance of observed groupings by testing whether the genetic diversity within each group was different from the pooled genetic diversity.

Analysis of similarity (ANOSIM) and Kruskal-Wallis test were done in mothur. ANOSIM was also used to compare the community composition (beta diversity) among sampling groups. Kruskal-Wallis test was used to examine significant differences in abundance of individual OTUs among groups. Results are discussed at the taxonomic level of order, due to short sequence reads [43]. All analyses were done at *α* = 0.05.

Putative microbiota functions were predicted using PICRUSt [44]. OTUs were mapped to the Greengenes database version 13.5, and 85 % of genes were classified to a Tier 1 KO function. The weighted nearest sequenced taxon index (NSTI) scores averaged 0.056 ± 0.018, indicating a relatively good match to reference genomes (ideal NSTI ≤ 0.03). Functional predictions were assigned up to KO tier 2. Tier 1 KO were compared among fishes using one-way ANOVA and Tukey post hoc tests for wild and lab fishes separately. Student’s *t* test was used to compare KO between wild and lab fishes and between lake and river environments for common carp. Due to numerous significant differences among groups in tier 2 KO, data were visualized using PCA. Functional classifications of chitinases and vitamin B_12_ synthesis enzymes were compared between wild and laboratory-housed bighead carp using Student’s *t* test. All statistical analysis of functional data was done using JMP, Version 10 (SAS Institute Inc., Cary, NC).

## Results

### Diversity and richness

**Fig. 1:**
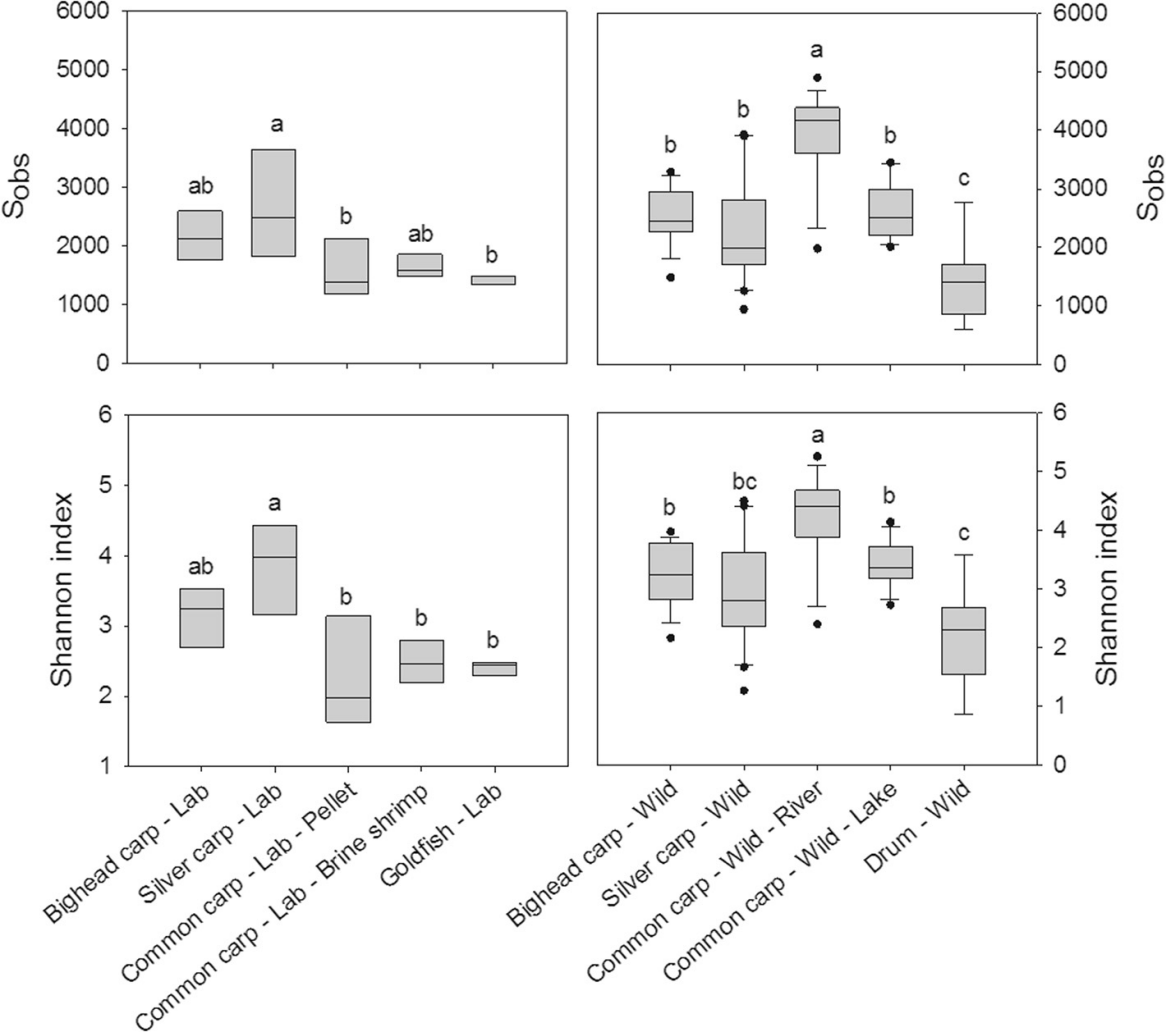
Diversity and observed richness of microbiomes across species and habitats. Groups indicated with the *same letter* are not significantly different at *α* = 0.05 using Tukey post hoc test

A total of 14,651 OTUs were identified across all 102 samples, with a mean coverage (estimate of total diversity that has been sampled) of 99 % ± 0.2 % (mean ± standard deviation) which ranged from 98 to 100 %. Observed species richness (S_obs_) and alpha diversity, calculated using Shannon index, differed significantly among species (*p* < 0.0001, Fig. 1). Among lab fishes, silver carp had the greatest mean richness and diversity but were not significantly different than bighead carp (*p* > 0.05). Among wild fishes, common carp from the river environment had greater richness and diversity than did the other wild fishes examined (*p* ≤ 0.05). Student’s *t* test comparison between the gut microbiome of river and laboratory-housed invasive carps showed that common carp exhibited significantly higher richness (*p* ≤ 0.0001) and diversity in wild fish (*p* = 0.002). Diversity was higher in captive silver carp relative to wild fish (*p* = 0.04), but richness did not differ (*p* = 0.46). Bighead carp did not show differences in either metric (*p* > 0.05).

**Fig. 2:**
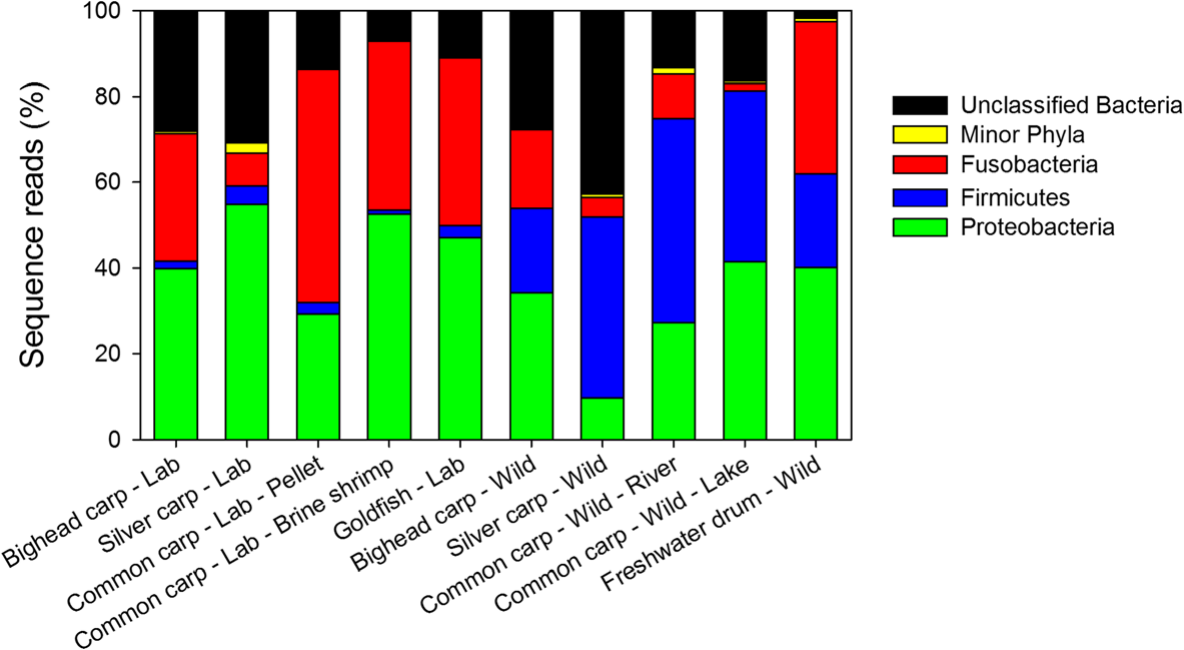
Taxonomic composition of microbial communities across environments. Phylum level relative abundance of fecal microbiome of each group averaged across individuals. *Designations* refer to species and habitat

Members of the phyla *Proteobacteria*, *Firmicutes*, and *Fusobacterium* dominated the gut microbiomes, comprising 76.9 % of total reads (Fig. 2). A portion (22.3 %) of all reads could not be classified to specific phyla, and other phyla comprised < 1 % of total reads. The most abundant orders included *Clostridiales*, *Fusobacteriales*, *Aeromonadales*, *Enterobacteriales*, *Xanthomonadales*, and *Vibrionales*. All other orders made < 1 % of total reads. The proportion of OTUs for each species at the taxonomic rank of family can be found in Additional file 1: Table S1 for families that had > 1 % of total reads.

### Distinct and shared OTUs

There were many OTUs that differed among fish species within and between environments. Of the 13,793 OTUs identified in wild fish, 76.6 % differed in abundance across species (*p* ≤ 0.05). However, OTUs that differed significantly among species made up 94 to 99 % of total reads per species. Among the laboratory fishes examined, only 20.5 % of the 7262 OTUs identified varied among species, making up 78 to 86 % of reads.

**Fig. 3:**
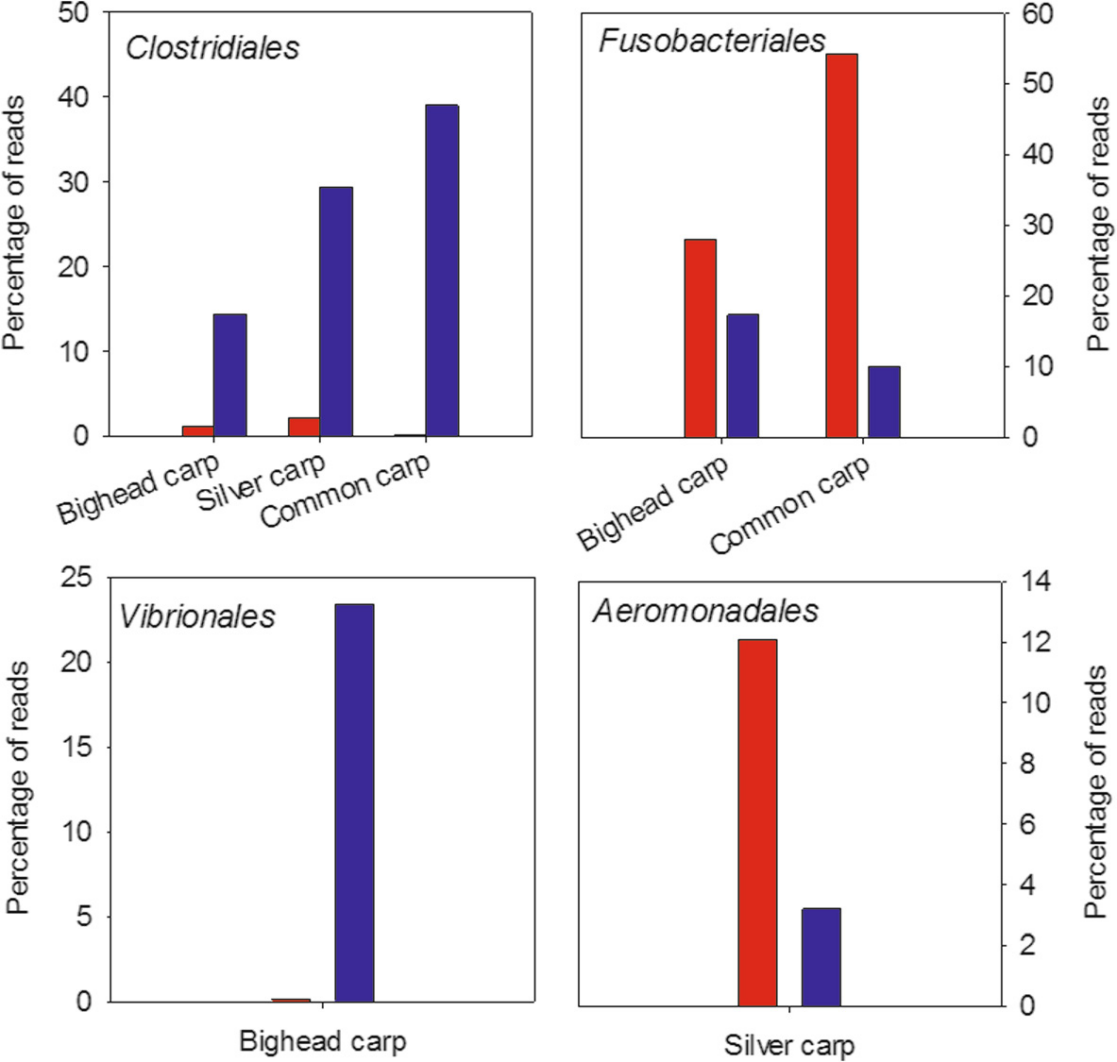
Order-level classification of OTUs that varied significantly between lab (*red*) and wild (*blue*) fish by Kruskal-Wallis test at *α* = 0.05. Unclassified bacteria and orders with less than 10 % of reads are not shown

There were systematic differences in the order of OTUs that differed between wild and laboratory-housed fish of the same species (Fig. 3). Significantly different OTUs within *Clostridiales* were more abundant in wild fish of all species, whereas OTUs within *Fusobacteriales* were more abundant in captive bighead and common carp. A large number of *Vibrionales* differed between wild and captive bighead carp, with higher abundance in wild fish. Finally, OTUs within *Aeromonadales* were higher in laboratory-housed silver carp.

Core gut microbiota must be present within a fish species across habitats [45]. We examined the OTUs that were present across wild and lab fish of the same species, and five OTUs emerged as the dominant bacteria of the core gut microbiota of invasive carps (Table 2). These bacteria were present at abundances of at least 1 % in all habitats, and they comprised, on average, approximately 40 % of the total fecal microbiome of invasive carps. There were three OTUs that were common across all invasive carps, and they were classified to the orders *Aeromonadales*, *Xanthomonadales*, and *Fusobacteriales*. One OTU unclassified at the phylum level was common to silver and bighead carps, whereas another unclassified OTU was prevalent in bighead carp only. We used the sequence of the closest Blast_*N*_ match for both unclassified bacterial OTUs to search against the 16S ribosomal RNA database. Both sequences returned close matches to species within the *Bacteroidetes* phylum. For common carp, there was no single OTU that was prevalent, yet specific, to that species.

**Table 2:** Mean relative abundance of OTUs in invasive carp species across environments

Order	Description, similarity and accession number, and source of closest NCBI Blast_*N*_ match	Abundance		
		Bighead carp	Silver carp	Common carp
Unclassified	Uncultured prokaryote, 98 % identity to KC601630, Asian seabass intestine	6.2	19.4	NA
*Aeromonadales*	*Psychrobacter* sp., 100 % identity to EU753148, marine intertidal flat	4.3	7.1	10.0
*Xanthomonadales*	Uncultured clone, 98 % identity to KM312603, earthworm gut	2.7	5.3	6.2
*Fusobacteriales*	*Cetobacterium* sp., 98 % identity to KM85610, Zebrafish intestine	21.8	5.4	23.8
Unclassified	Uncultured prokaryote, 98 % identity to KC601623, Asian seabass intestine	4.3	NA	NA
Sum		39.3	37.2	40.0

OTUs with < 1 % prevalence across all groups and species are not reported

*NA* not applicable

### Differences in community composition

**Fig. 4:**
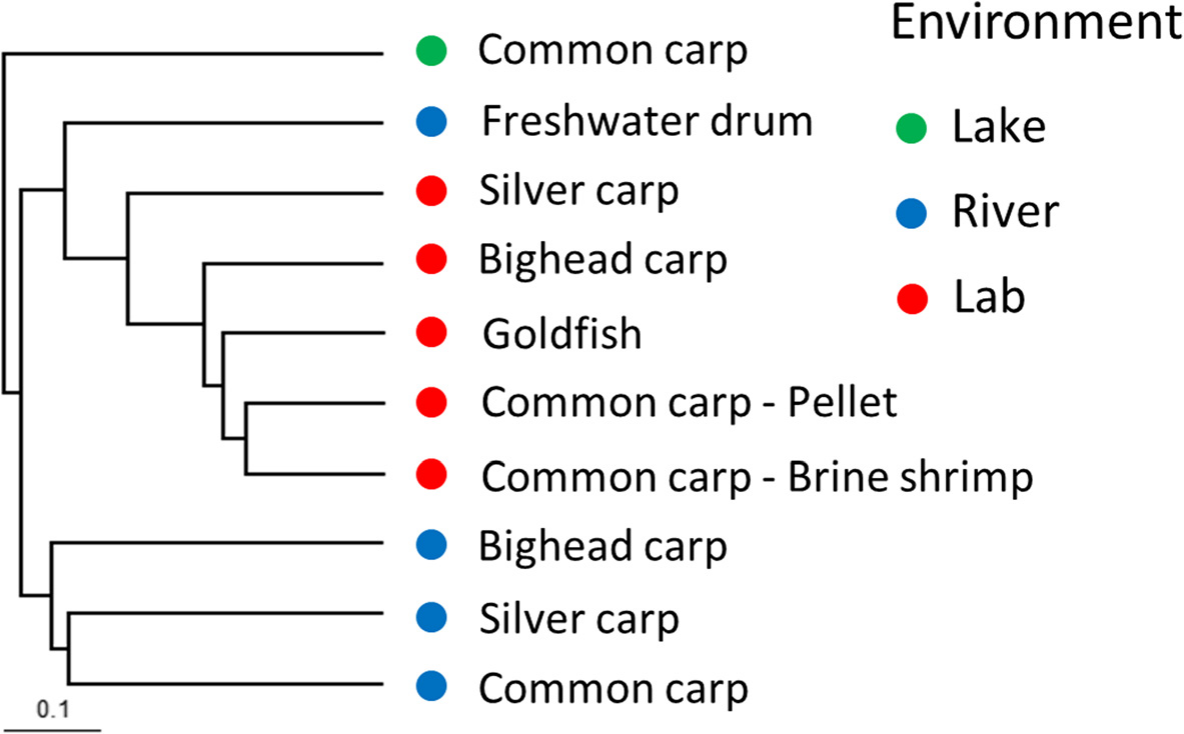
Hierarchical clustering of species-environment groups based on Bray-Curtis dissimilarity indices using the Unweighted Pair Group Method (UPGMA)

**Fig. 5:**
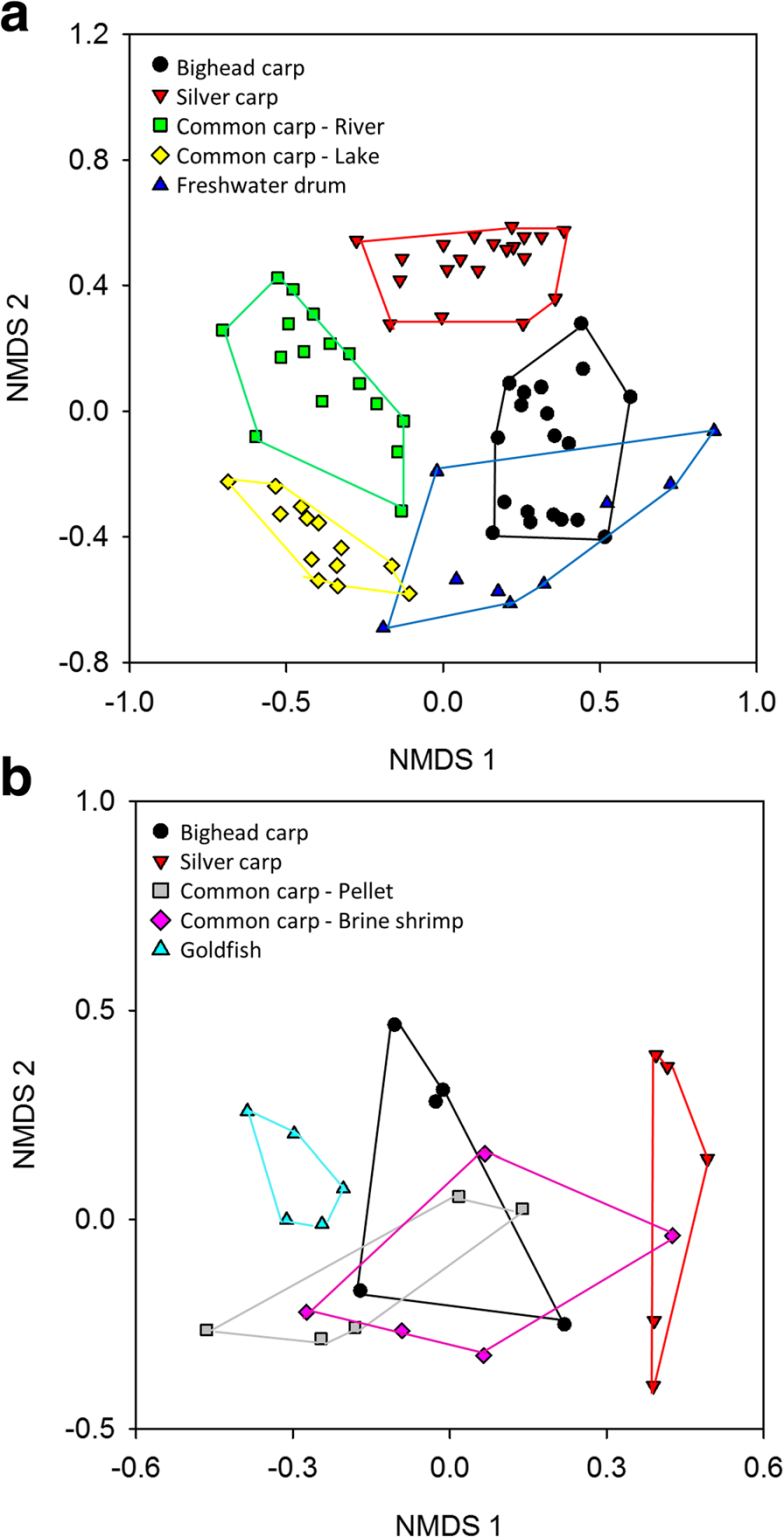
Ordination of fecal microbiomes of **a** wild and **b** laboratory fish. Non-metric multidimensional scaling was used, and distance was based on Bray-Curtis dissimilarity. The *R* ^2^ for plots of wild and laboratory fish communities was 0.67 and 0.77, respectively. Convex hulls connect individuals from the same group

Hierarchical clustering showed that bacterial communities clustered primarily by environment (Fig. 4). Non-metric multidimensional scaling (NMDS) of wild fishes showed separation of most fish by species, and the lowest stress value was 0.29 with an *R*^2^ of 0.67 (Fig. 5). Although the distribution of freshwater drum slightly overlapped with bighead and lake common carp, all groupings were significantly different (AMOVA, *p* < 0.001). The NMDS analysis was able to capture slightly more variability within the data for lab fishes (stress value = 0.23, *R*^2^ = 0.77). The distribution of bighead and common carp that were fed pellets and brine shrimp overlapped (Fig. 5), and these communities did not have significantly different grouping (AMOVA, *p* > 0.05). The gut bacterial communities of silver carp and goldfish did not group with other lab fishes (Fig. 5), and both species were significantly different from other lab fish species (AMOVA, *p* ≤ 0.05).

ANOSIM showed significant differences among groups in community composition and abundance (beta diversity). All wild fish species had distinct microbial communities (*p* < 0.0001), and the gut microbiota between wild and lab fish of the same species were different (*p* < 0.0001). For lab fishes, however, fewer differences were observed. Bighead carp were not significantly different from common carp fed pellet feed (*p* = 0.08) or brine shrimp (*p* = 0.18) diet. Diet did not change the bacterial community composition of common carp (*p* = 0.30). All other comparisons among lab fish were significant at *α* = 0.05.

### Functional analysis

**Fig. 6:**
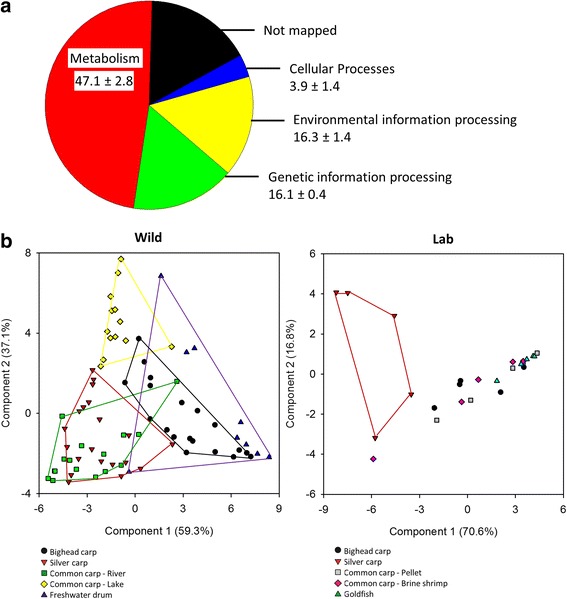
PICRUSt classification of KEGG Orthologies (KO). **a** Tier 1 KO functions across all groups for functions greater than 1 % of total gene counts for each KO. Average and standard deviation across species are shown below labels. **b** Principal components analysis of Tier 2 KO functions for wild and lab fish groups. Convex hulls connect individuals from the same group, but they are not shown for some lab fish due to overlapping distribution

Functional assignments were predicted from microbial community composition using PICRUST, which revealed differences in predicted microbial function across species and environments. The majority of predicted tier 1 KEGG Orthology (KO) were in the functional category of metabolism (Fig. 6).

Comparisons of species within wild and lab environments were done using principal components analysis (PCA) due to numerous significant differences in tier 2 KO functions. The PCA components 1 and 2 explained a large proportion in the variation of the data (Fig. 6; wild fish = 89.9 %, lab fish = 88.4 %). Although there was overlap in the distribution of points representing the inferred function of the microbiota of wild fish, captive fish had more similarity in the predicted function of the microbiome than among wild fish. All lab fish, apart from silver carp, had overlapping distributions of the graphical representation of inferred microbiome functional data (Fig. 6). Transcription was weighted heavily in axis 1 of both wild and lab fish, indicating that this category is particularly important in the differentiation of gene functions among fish species (Table 3).

**Table 3:** Axis loadings for principal components analysis of tier 2 KO functions

Wild fish		Laboratory-housed fish	
Axis 1	Axis 2	Axis 1	Axis 2
Positive	Positive	Positive	Positive
o Xenobiotics biodegradation and metabolism o Metabolism of terpenoids and polyketides o Lipid metabolism	o Signal transduction o Cellular processes and signaling o Metabolism of other amino acids	o Carbohydrate metabolism o Nucleotide metabolism o Replication and repair	o Lipid metabolism o Metabolism of terpenoids and polyketides o Xenobiotics biodegradation and metabolism
Negative	Negative	Negative	Negative
o Membrane transport o Transcription	o Carbohydrate metabolism o Translation	o Transcription o Cellular processes and signaling	o Amino acid metabolism o Enzyme families

Factors shown had the five largest loadings and were ≥ 1 % of inferred function

In a comparison of wild and laboratory-housed fish of the same species, there were no differences in tier 1 KO functions that were consistent across the three species. Comparisons of the proportion of proposed function for five vitamin B_12_ biosynthesis proteins between wild and lab fishes showed some differences. For instance, there was a higher proportion of vitamin B_12_ biosynthesis for two enzymes in wild silver carp and all five in common carp (*p* ≤ 0.05). Chitinase functional classification was not different (*p* = 0.20) between wild and laboratory bighead carp.

## Discussion

### The role of diet and environment in shaping the fish microbiome

Bacterial community structure and function in the guts of carps was strongly affected by environment. There were large differences between laboratory-housed and wild fish of the same species and between common carp and lake and river environments. Many of the differences between wild and lab fishes can likely be partially explained in the context of diet, and the three taxonomic orders that varied most between wild and lab fish were *Clostridiales*, *Fusobacteriales*, *Vibrionales*, and *Aeromonadales*.

*Clostridiales*, which were more abundant in all wild-invasive carp species compared to those reared in the laboratory habitat, are associated with the degradation and metabolism of carbohydrates, specifically sugars [46, 47]. *Clostridiaceae* also aid in glucose fermentation as has been shown in earthworm guts [48] and are responsible for producing short chain fatty acids in vertebrates. Prebiotic arabinoxylan oligosaccharides have been shown to increase *Clostridium* spp. in sturgeon [23, 49]. Therefore, greater plant matter intake might increase the proportion of *Clostridiales*. Despite this supposition, however, both silver and bighead carps in the lab were fed a diet composed primarily of cyanobacteria and green algae. Moreover, feeding preference for cyanobacteria was similarly confirmed for wild silver carp [29]; thus, differences in plant matter intake cannot solely explain patterns of abundance of the *Clostridiales*.

An alternative explanation is that feed timing or availability in the wild carps resulted in changes in their gut microbiota. Transgenic common carp exhibited higher food intake and growth rate, which was associated with a greater proportion of *Clostridiales* in the gut microbiome [50]. In animals and humans, this increase is thought to be due to enhanced production of the short chain fatty acids acetate, butyrate, and propionate [50]. Although lab fish were fed to satiation, they ate only once daily during the daytime. Wild-invasive carps, in contrast, are able to consume food over a larger time frame, and they feed primarily at night [7, 51]. There is limited information on how food availability or timing affects fish gut microbiota; however, a study on Asian seabass showed starvation-induced changes in proportion of *Bacteroides* [52]. While members of the genus *Bacteroides* were not abundant in invasive carps, it is unclear what effect food limitation might have on invasive carp species, and this topic warrants further study.

Laboratory-housed carp had a much higher proportion of *Fusobacteriales* compared to wild fish. Over 95 % of *Fusobacteriales* were classified to the genus *Cetobacterium. Cetobacterium somerae*, a bacterium within the order *Fusobacteriales*, is a common and widely distributed species within the guts of freshwater fishes, and its prevalence is negatively correlated with dietary availability of vitamin B_12_ (cobalamin) [53, 54]. Hence, the main role of *Cetobacterium somerae* in the fish gut is assumed to be synthesis of vitamin B_12_ [53, 54]. The relative proportion of vitamin B_12_ within each food type in this study is not known, so we are unable to make comparisons in the relative proportion of *Fusobacteriales* across diets. Bighead and silver carp can obtain B_12_ from algal food sources. Cyanobacteria are capable of synthesizing vitamin B_12_, and although many eukaryotic algae do not directly synthesize B_12_, they have symbioses with B_12_-producing bacteria [55, 56]. As wild common carp eat a significant amount of detritus, they may also satisfy their B_12_ requirements through consumption of bacteria [2]. However, we found that wild common carp and silver carp had a higher proportion of vitamin B_12_ functional classifications than captive fish with a higher abundance of total *Fusobacteriales*.

For bighead carp, the bacterial order *Vibrionales* exhibited the greatest difference in abundance between wild and laboratory environments. These bacteria are may be dietary in origin. *Vibrionales* are associated with exoskeletons of zooplankton [57], a major food source for bighead carps. Although silver carp consume zooplankton, such as cladocerans and copepods, these organisms make up a smaller proportion of the silver carp diet compared to bighead carp [58]. Dietary chitin has not been conclusively shown to increase the proportion of chitinase-producing bacteria in fish guts in aquaculture applications [59]; however, chitinase-producing bacteria have been isolated from many fishes, including common carp [12]. We found no difference in the proportion of chitinase function between wild and captive bighead carp, further indicating that *Vibrionales* were not contributing significantly to chitinase activity in the gut.

The fecal microbiota of common carp from lake and river habitats were different with respect to bacterial community structure, richness, and diversity. Generally, all these parameters were greater in river fish than those dwelling in lakes. Common carp exhibit similar diets in both lake and river environments. The primary component of the stomach contents of both lake and river carp is detritus, while seeds and invertebrates make up the majority of the remaining contents [1, 2, 60]. Therefore, differences are likely due to environment-specific factors rather than diet alone.

In wild fishes, patterns in relative abundance of bacterial phyla reflected feeding preferences. While silver carp and bighead carp are both filter feeders, phytoplankton makes up a larger proportion of the silver carp diet [7]. Common carp are omnivores, and their diet includes detritus, invertebrates, and plant matter [2], whereas freshwater drum are carnivorous [61, 62]

We found no consistent trends in phyla across trophic level, possibly due to the large variation in common carp between lake and river habitats.

We found a much greater proportion of unclassified bacteria than in previous studies [22, 29]. When considering the cumulative frequency of unclassified reads, most were present in high abundance (Additional file 2: Figure S1). Moreover, abundant sequences that were unclassified at the phylum level were distributed across species. For example, the three most abundant sequences are very prevalent across species (Additional file 3: Figure S2), and they are present in 88 to 99 % of individual fish sampled. While removal of sequence reads that were ≤ 10 in abundance decreased overall diversity, results of statistical analyses and beta diversity indices remained the same. Taken together, the abundance of bacteria unclassified at the phylum level and their distribution across fish species indicates that there is considerable diversity in the fish gut that is uncharacterized.

There are differences in the microbial community structure observed in the present study to previous studies on wild carps. For example, we did not observe a significant proportion of *Bacteroidetes* or *Actinobacteria* in the silver carp microbiome, as was observed by Ye et al. [29]. The differences may be due to differences in the geographic location of the fish sampled or gut samples were taken. Generally, fish in the present study were sampled further north, and fecal material was collected by stripping rather than dissecting. Li et al. [22] found a predominance of *Proteobacteria* and comparatively few *Firmicutes* in bighead carp in their native range. However, samples were collected in the winter, when fish were under starvation conditions, which is known to alter gut microbial composition [52].

The distribution of phyla in captive carps was similar to that found in previous studies, with a few exceptions. In common carp, we found nearly 50 % of phyla classified to *Fusobacteria*, similar to van Kessel et al. [33]. The proportion of *Fusobacteria* and *Firmicutes* in bighead carps was similar to Li et al.’s [22]. However, we found a smaller proportion of *Bacteroidetes* than both studies.

### Effect of captivity on the fish microbiome

Although patterns in microbiota in wild fish appeared to be linked with feeding preferences, we found that diet had little effect on fecal microbiome in a lab setting. For example, there was no difference in microbial community of common carp fed pellet or brine shrimp by any statistical measure of community composition. Previous studies have shown that diet can influence the fish gut microbiota by introduction of prey-associated microbes [26]. However, we do not have any data in this study on the microbes present in the foods ingested by fishes or the microbiome of the surrounding habitat. Intraspecific changes in the gut microbiome may also result from alteration in food metabolism, as shown in Eastern African cichlids and surgeon fishes [25, 63]. However, changes in response to diet can be slight [24] and may depend on species [64] or ecotype [28]. We also found that bighead and silver carp had different microbiota, despite being fed a similar algal feed mixture. Our results support those of Li et al. [65] who found that paddlefish and bighead carp reared in the same pond had distinct intestinal microbiota.

In lab fishes, species differences were not as pronounced as for wild fishes. For example, fewer OTUs differed in their relative abundance among lab fish species, and fewer differences among bacterial communities were observed. Temperature is a driving force in the biology and behavior of poikilotherms, such as fishes [66]. In laboratory or captive environments, fishes are not exposed to diurnal cycles in water temperature, nor is there the level of habitat complexity that is found in the natural environment. Differences in habitat use between closely related species of cichlids within a lake were associated with differences in microbiota [67]. Thus, differences in microbiota among species in the wild are likely due to a combination of the effects of diet, habitat usage, temperature, physiology, and taxonomy. Thus, the homogeneous and homeostatic environment of the laboratory might modulate behavioral effects on microbiota, such as habitat usage.

Only common carp exhibited a difference in alpha diversity between wild and captive fish, with lower diversity in captive fish. The effect of captivity on gut microbiome diversity may be species-dependent. Captivity did not reduce the diversity of zebrafish gut microbiome [30]. However, captivity dramatically reduced in cichlid fish [25] and mummichog [68].

Our results indicate a striking effect of environment on the fecal microbiome of invasive carps and, in particular, a dramatic effect of captivity. In a previous study, few differences between the gut microbiota of wild and lab populations of zebrafish were observed [30]. But this may be due to rearing practices as the wild zebrafish were held for approximately 1 month under laboratory conditions prior to sampling, and this time frame was previously shown to alter the gut microbial community of silver carp [29]. Several studies have observed differences in microbiota of fishes in captive versus wild environments that mirror our findings. In a study of pond-reared and wild grass carp, *Fusobacteria* was more prevalent in pond fish [69]. A predominance of *Fusobacteria* was also found for grass carp, crucian carp, and bighead carp held in a rearing pond and fed a commercial feed [22]. Thus, we support the contention that environment shapes the fish gut microbiome and that a true understanding of their gut microbiota needs to come from wild-caught fish.

### The core microbiome of invasive carps

Ringø and Birkbeck [45] enumerated five criteria required to be considered core gut microbiota in fishes: (1) they must be present in healthy individuals, (2) they colonize the gut at early life stages and persist throughout the lifespan of the fish, (3) they are found in both wild and cultured fish populations, (4) they are able to grow anaerobically, and (5) they are associated with the stomach, foregut, or hindgut. We identified five OTUs that comprise a large proportion of the core microbiome of silver, bighead, and common carps. These OTUs satisfied criteria 1, 3, and 5, but additional experiments would be needed to evaluate whether these OTUs satisfy all criteria.

Previous studies have shown that the proportion of bacteria that make up the core microbiome is variable [68]. In zebrafish, 21 OTUs comprise the core microbiome [30]. In rainbow trout, the core microbiota makes up over 80 % of the total community, and it is resistant to environmental factors [24]. Future work is needed to further identify and characterize the core microbiota of carps. In addition, studies should assess when these bacteria initially colonize the gut and their degree of persistence over time. Moreover, due to the disproportionate abundance of some OTUs in bighead and silver carp, the potential for utilizing core microbiota to identify the presence of these invasive fishes in water bodies through the identification of their associated microbes be explored [70, 71].

## Conclusions

Our results indicate that environment is an important factor controlling invasive carp fecal microbiota. We draw this conclusion from the difference between lab and wild fishes of the same species and the difference between lake and river habitats for common carp. Diet may partially explain some patterns in phyla abundance for wild fishes. However, in laboratory-housed fishes, diet did not exert a strong effect on fish gut microbiota, rather, fish species was the factor controlling differences among lab fishes. Future studies are needed to tease apart the multitude of factors which potentially control fish microbiota in wild populations. The mechanisms underlying differences in microbiota between lake- and river-dwelling common carp, for example, are not known. Due to the role of environment in shaping the microbiome, source-tracking markers for specific fish species should be developed from large representative samples of individuals from different geographical areas and habitat types. Studies on laboratory populations of fishes should be interpreted with caution, as lab fish have distinct microbiome structure from those of wild fishes, a pattern which is apparent, but not well characterized, in other studies. Invasive carps have a core fecal microbiome comprised primarily of five bacterial species, which make up approximately 40 % of the total fish gut microbiome. Future research is needed on the specific functional role of these organisms within the carp fecal microbiome. In addition, potential for augmenting these microbes for aquaculture applications or species detection or control should be evaluated.

## Abbreviations

AMOVA, analysis of molecular variance; ANOVA, analysis of variance; IACUC, Institutional Animal Care and Use Committee; KO, KEGG Orthology; NMDS, non-metric multidimensional scaling; NSTI, nearest sequenced taxon index; OTU, operational taxonomic unit; PCA, principal components analysis; RDP, Ribosomal Database Project; UPGMA, Unweighted Pair Group Method

## Additional files

Additional file 1: Table S1. Percentage abundance of OTUs at the family level greater than 1 % of total reads. (DOCX 14 kb) Additional file 2: Figure S1. The cumulative proportion of sequences unclassified at the phylum level with increasing frequency. (TIF 131 kb) Additional file 3: Figure S2. Percentage of reads across samples for the three most abundant sequences that are unclassified at the phylum level. (TIF 209 kb)
