# Supplementary material for: Environment shapes the fecal microbiome of invasive carp species
Source: Microbiome. 2016 Aug 12;4:44. doi: 10.1186/s40168-016-0190-1 (PMC4981970; doi:10.1186/s40168-016-0190-1)
Supplement: Additional file 1: Table S1. — Percentage abundance of OTUs at the family level greater than 1 % of total reads. (DOCX 14 kb) [file 40168_2016_190_MOESM1_ESM.docx]

Table S1. Mean relative abundance of OTUs at the family level greater than 1% of total reads.

| Family | Bighead carp - Lab | Silver carp - Lab | Common carp - Lab - Pellet | Common carp - Lab - Brine shrimp | Goldfish - Lab | Bighead carp - Wild | Silver carp - Wild | Common carp - Wild - River | Common carp - Wild - Lake | Freshwater drum - Wild |
| --- | --- | --- | --- | --- | --- | --- | --- | --- | --- | --- |
| *Fusobacteriaceae* | 29.7 | 7.6 | 54.4 | 39.3 | 39.1 | 18.4 | 4.6 | 10.2 | 1.8 | 35.2 |
| *Clostridiaceae 1* | 0.0 | 0.3 | 0.0 | 0.0 | 0.0 | 9.0 | 23.0 | 17.7 | 9.2 | 1.0 |
| *Aeromonadaceae* | 5.1 | 12.1 | 6.9 | 10.6 | 2.6 | 4.0 | 3.2 | 5.4 | 19.3 | 12.1 |
| *Peptostreptococcaceae* | 1.1 | 1.7 | 0.3 | 0.0 | 0.2 | 1.9 | 5.4 | 19.5 | 3.3 | 4.8 |
| *Enterobacteriaceae* | 12.0 | 4.7 | 6.7 | 9.4 | 21.2 | 1.1 | 0.9 | 0.8 | 0.5 | 20.9 |
| *Xanthomonadaceae* | 3.2 | 10.2 | 4.1 | 9.5 | 12.8 | 2.6 | 1.7 | 4.0 | 9.1 | 3.3 |
| *Vibrionaceae* | 0.3 | 1.3 | 0.0 | 0.0 | 0.1 | 23.4 | 0.1 | 0.1 | 1.8 | 0.1 |
