# Supplementary figures and images for: Environment shapes the fecal microbiome of invasive carp species
Source: Microbiome. 2016 Aug 12;4:44. doi: 10.1186/s40168-016-0190-1 (PMC4981970; doi:10.1186/s40168-016-0190-1)

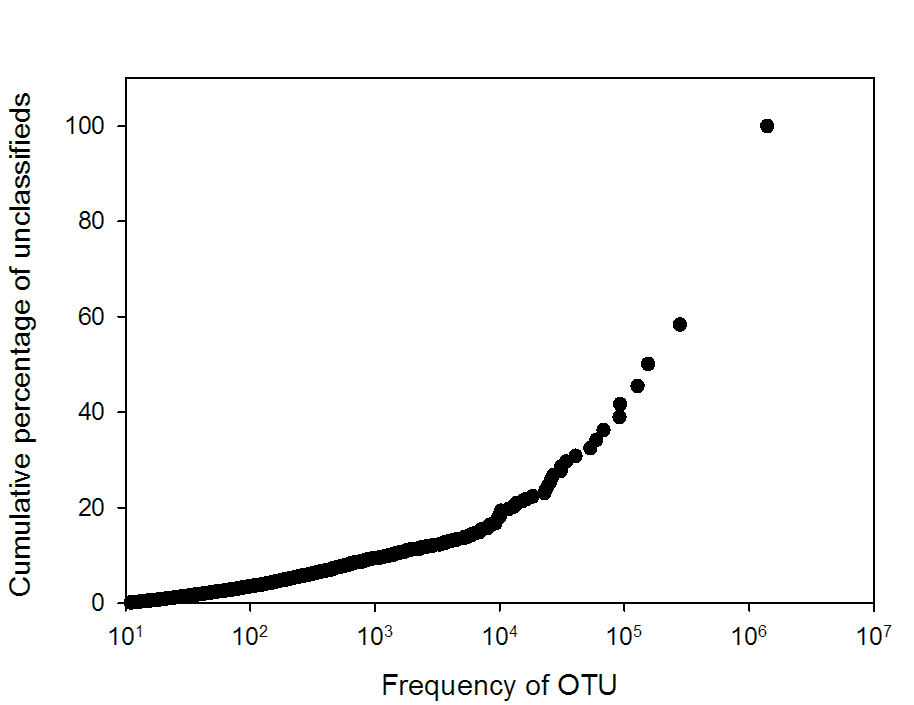

Supplement: Additional file 2: Figure S1. — The cumulative proportion of sequences unclassified at the phylum level with increasing frequency. (TIF 131 kb) [file 40168_2016_190_MOESM2_ESM.tif]

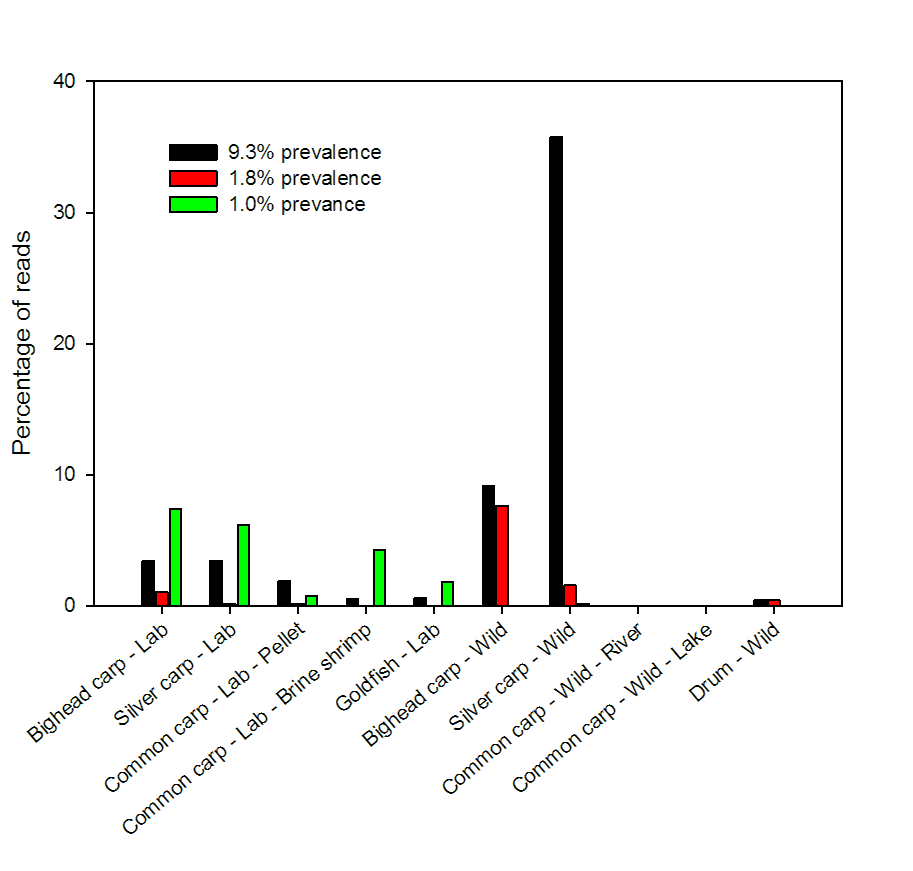

Supplement: Additional file 3: Figure S2. — Percentage of reads across samples for the three most abundant sequences that are unclassified at the phylum level. (TIF 209 kb) [file 40168_2016_190_MOESM3_ESM.tif]
